# Patterns of Use and Withdrawal Syndrome in Dual Cannabis and Tobacco Users (DuCATA_GAM-CAT): Protocol for a Mixed Methods Study

**DOI:** 10.2196/58335

**Published:** 2024-09-19

**Authors:** Judith Saura, Ariadna Feliu, Marta Enríquez-Mestre, Marcela Fu, Montse Ballbè, Yolanda Castellano, Margarida Pla, Nathalia Rosa, Petia Radeva, Elena Maestre-González, Carmen Cabezas, Joan Colom, Josep M Suelves, Silvia Mondon, Pablo Barrio, Magalí Andreu, Antònia Raich, Jordi Bernabeu, Jordi Vilaplana, Xavier Roca Tutusaus, Joseph Guydish, Esteve Fernández, Cristina Martínez

**Affiliations:** 1 Tobacco Control Unit, Cancer Control and Prevention Program Institut Català d’Oncologia WHO Collaborating Center On Tobacco Control Barcelona Spain; 2 Cancer Control and Prevention Group Institut d’Investigació Biomèdica de Bellvitge-IDIBELL l’Hospitalet de Llobregat Spain; 3 CIBER en Enfermedades Respiratorias, CIBERES Instituto Salud Carlos III Madrid Spain; 4 Department of Clinical Sciences School of Medicine and Health Sciences University of Barcelona Barcelona Spain; 5 Department of Public Health, Mental Health, and Maternal and Child Health Nursing School of Nursing - Bellvitge Campus Universitat de Barcelona Barcelona Spain; 6 Addictions Unit Institute of Neurosciences Hospital Clínic de Barcelona Barcelona Spain; 7 Campus Docent San Joan de Deu Sant Boi de Llobregat Spain; 8 Department of Medicine and Life Sciences Pompeu Fabra University (UPF) Barcelona Spain; 9 Department of Mathematics and Computer Science University of Barcelona Barcelona Spain; 10 School of Nursing Faculty of Medicine and Health Sciences University of Barcelona Barcelona Spain; 11 Government of Catalonia Public Health Secretariat Barcelona Spain; 12 Public Health Agency of Catalonia Barcelona Spain; 13 Universitat Oberta de Catalunya Barcelona Spain; 14 Addictions Unit, Psychiatry Department Institute of Neurosciences Hospital Clínic de Barcelona Barcelona Spain; 15 Mental Health Department Althaia Xarxa Assistencial Universitària Manresa Spain; 16 Serra Húnter Fellow Computer Science Department Universitat de Lleida Lleida Spain; 17 Addictive Behaviors Unit Psychiatry Department Hospital de la Santa Creu i Sant Pau Barcelona Spain; 18 Institut d'Investigació Biomèdica Sant Pau (IIB Sant Pau) Barcelona Spain; 19 Philip R. Lee Institute for Health Policy Studies University of California San Francisco San Francisco, CA United States

**Keywords:** cannabis, tobacco, substance abuse, withdrawal symptoms, mobile phone, protocol, addiction, pattern use, withdrawal syndrome, mixed method, participatory, qualitative study, focus groups, cannabis use disorder, clinicians, researchers, predictive analysis

## Abstract

**Background:**

Approximately 1 in 6 cannabis users develop a cannabis use disorder (CUD) and the odds increase to 1 in 2 for daily users.

**Objective:**

The Dual use of Cannabis and Tobacco Monitoreing through a Gamified Web app (DuCATA_GAM-CaT) project aims to identify cannabis-tobacco patterns of use and withdrawal symptoms among individuals with CUD who are attending substance abuse programs.

**Methods:**

The project uses a mixed methods approach consisting of 3 studies. First, a participatory qualitative study involves focus groups comprising individuals with CUD, clinicians, project researchers, and an expert gamification company to co-design a gamified web app. Second, a longitudinal prospective study to follow up individuals over 6 weeks with CUD attending substance abuse programs . Participants report their cannabis-tobacco usage patterns, type and frequency of tobacco use, nicotine dependence, withdrawal symptoms, psychoemotional factors, and motivation to quit both substances. Predictive analysis techniques are used to analyze clinical, demographic, psychological, and environmental data to predict the probability of achieving abstinence. Third, homogeneous focus groups to explore participants’ experiences during their CUD treatment.

**Results:**

By June 2024, the project had completed the first study, defining eligible cannabis user profiles, developed the initial web app prototype, and initiated recruitment across 10 centers, with 74 participants enrolled, aiming to reach 150 participants in total.

**Conclusions:**

All participants are required to provide informed consent, and their information is kept confidential and anonymized following confidentiality rules. The research team is committed to disseminating the results obtained to professional and patient groups, as well as informing public health agents, to positively influence political and social decision makers and design programmers. Additionally, we aim to prioritize the publication of the results in high-impact journals specialized in drug abuse, public health, and health care services research.

**Trial Registration:**

ClinicalTrials.gov NCT05512091; https://clinicaltrials.gov/study/NCT05512091

**International Registered Report Identifier (IRRID):**

DERR1-10.2196/58335

## Introduction

### Cannabis and Tobacco Use

Cannabis has emerged as the most widely used illegal drug globally [1]. In Spain, it also holds the status of the most commonly used illegal drug. According to the latest survey conducted in Spain in 2019, 37.5% of adults aged 15-64 years have experimented with cannabis at some point in their lives, with 10.5% reporting usage in the last month (men: 11.4% and women: 4.7%) [2]. Furthermore, approximately 1 in 6 cannabis users develop a cannabis use disorder (CUD) and the odds are increasing to 1 in 2 among daily users [2].

In Western countries, cannabis use is commonly intertwined with tobacco**,** resulting in a significant prevalence of combined usage that continues to rise [3]. For instance, in the United States, the daily use of both substances remained stable over the years from 2005 to 2014, with a usage percentage of 6%. This proportion is higher than that of individuals who consume only cannabis without tobacco, which is around 2% [4]. However, it is important to note that dual cannabis and tobacco use, or co-use, can manifest in various patterns such as (1) concurrent use, where both substances are consumed simultaneously in the form of joints, which is the most prevalent form of cannabis use in Europe [3,5,6]; (2) sequential concurrent use, involving the use of cannabis followed by tobacco or vice versa; (3) asynchronous concurrent use, characterized by cannabis and tobacco usage within the past month but not necessarily during the same instance of consumption; and (4) exclusive cannabis use, where tobacco is not involved [4].

Moreover, dual cannabis-tobacco use entails a nuanced description of dependence diagnosis for both substances collectively and individually, alongside the emergence of withdrawal symptoms, particularly when cannabis consumption is reduced or halted [7-9]. Cannabis withdrawal symptoms typically manifest within 2 to 4 days after a reduction or cessation of consumption, peak during the first week, and may persist for up to 4 weeks [10]. The cannabis withdrawal syndrome is recognized and included in the *DSM-5* (*Diagnostic and Statistical Manual of Mental Disorders* [Fifth Edition]) [11]. It is characterized by symptoms such as irritability, anger or aggression, nervousness or anxiety, sleep difficulties (eg, insomnia and disturbing dreams), decreased appetite or weight loss, restlessness, depressed mood, and at least one of the following physical symptoms causing significant discomfort: abdominal pain, shakiness or tremors, sweating, fever, chills, or headache. The symptoms can be assessed using the “Cannabis Withdrawal Checklist” scale. While this scale has been validated [12] and used primarily in English-speaking populations [13-16], its applicability elsewhere warrants consideration.

A meta-analysis has estimated that 47% of cannabis users experience withdrawal syndrome upon cessation, with its occurrence often linked to tobacco use [9]. Additionally, the severity of the cannabis withdrawal syndrome inversely affects the likelihood of successful cessation, with more severe symptoms correlating with lower rates of quitting [9]. Given that cannabis withdrawal syndrome frequently contributes to relapse [17], resulting in modest rates of abstinence maintenance, it is noteworthy that only 30% of individuals who undergo treatment manage to sustain abstinence during the initial month [18]. Various factors contribute to relapse, including the use of other psychoactive substances [19], psychological variables (emotions, social pressure, etc), perceptions of social norms, self-efficacy [20], and sociodemographic variables (sex, age, ethnicity, etc) [16].

### Cannabis and Tobacco Dependence Withdrawal Syndromes

**Figure 1:**
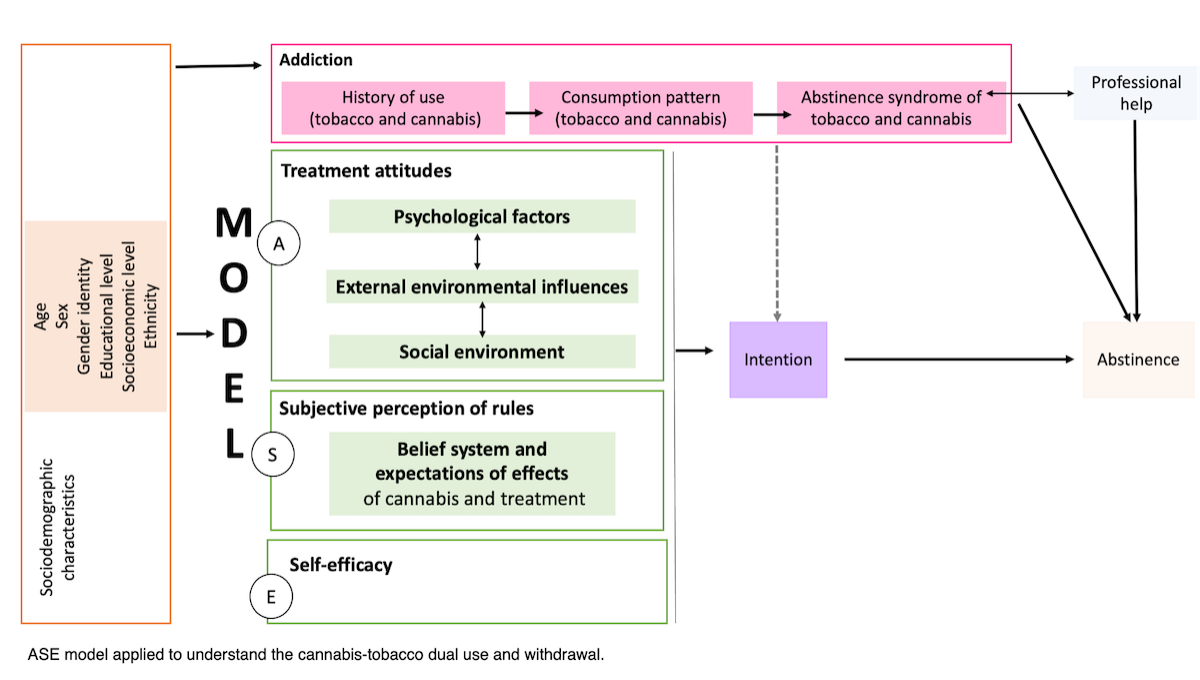
Logic model of the study. ASE: Attitudes, Social Influences, and Efficacy. de Vries H, Backbier E. Self-efficacy as an important determinant of quitting among pregnant women who smoke: the phi-pattern. Prev Med. 1994;23(2):167-174. [doi: 10.1006/pmed.1994.1023] [Medline: 8047522].

The limited number of studies conducted on individuals undergoing treatment for CUD who also use tobacco fail to provide sufficient insight into the interaction between the consumption patterns of both substances. Consequently, there exists a potential misclassification of withdrawal syndromes between cannabis and tobacco [10,21]. As a result, the influence of cannabis and tobacco dependence, along with their respective withdrawal syndromes, on cannabis use remains unclear, particularly in conjunction with other cognitive and environmental factors previously associated with substance use and cessation. Drawing from the social science literature and conceptual models that aid in understanding individual behaviors, we propose using the Attitudes, Social Influences, and Efficacy (ASE) model [20] to decipher the factors affecting cessation and maintenance processes. Therefore, we integrate this model [20] with individual attributes (such as sex, age, and socioeconomic status) and addiction characteristics (smoking pattern, dependence, etc) to unravel this intricate phenomenon (see Figure 1).

The lack of a detailed characterization of the influence of tobacco and cannabis use is partly due to the absence of techniques enabling real-time assessment, such as Ecological Momentary Assessment (EMA) [22]. While these powerful tools have been used to characterize and predict patterns among individuals with alcohol use disorder [23,24], they have yet to be used among cannabis users. Consequently, the combination of these tools could facilitate the characterization of the prevalence and patterns of dual cannabis-tobacco consumption, including a detailed description of the severity and types of symptoms experienced by users during cannabis withdrawal syndrome.

Consequently, due to limited knowledge regarding (1) how tobacco impacts cannabis withdrawal syndrome and (2) the effect of dual consumption on dropout probabilities during CUD treatment, we propose the Dual users of Cannabis and Tobacco (DuCATA) study. This mixed methods project encompasses 3 studies (qualitative, quantitative, and mixed methods) with a clinical and epidemiological focus, enabling the characterization and monitoring of consumption and withdrawal syndromes of cannabis and tobacco among individuals initiating treatment for CUD. The monitoring of dual cannabis-tobacco use and withdrawal symptoms will be conducted via a prospective follow-up of participants using a gamified web app [25] based on the EMA model [22,26] facilitating real-time collection of participants’ consumption and symptoms based on their daily experiences. Gamification, through the incorporation of gaming elements, encourages active user participation in data reporting, thereby enhancing adherence to the registry and improving data quality [25].

This knowledge bears significant implications for clinical practice and public health, as it will provide precise insights into the characteristics of dual cannabis-tobacco users, a profile increasingly prevalent in programs. Moreover, it will enable us to use and validate an instrument developed in April 2023 for monitoring cannabis withdrawal symptoms among the Spanish population and investigating nicotine dependence. Ultimately, we are confident that comprehending and describing the dual use of cannabis-tobacco will equip future studies with a tool to accurately assess this complex phenomenon and facilitate the design of tailored interventions.

### Study Objectives

#### Overview

The research aims of the “Characterization of the pattern of consumption and withdrawal syndrome of dual use of cannabis and/or tobacco in people with CUD who start cessation treatment: DuCATA study” can be described, indicating the studies in which each objective falls.

#### Objective 1 (First Study)

Determinate the treatment preferences of individuals with CUD, based on age (18-34 years and 35 years or older) and sex, for the design of a gamified web app.

#### Objective 2 (Second Study)

Examine the consumption patterns of cannabis and tobacco products among individuals seeking help to reduce and quit their cannabis use. This includes considering structural determinants of inequality (age, sex, social class or educational level, and territory) and clinical history (use of other substances, psychiatric comorbidity, etc), and identifying different typologies of cannabis-tobacco users, such as concurrent use, sequential use, asynchronous use, and exclusive cannabis use.

#### Objective 3 (Second Study)

Analyze changes in tobacco use during the cannabis treatment process based on the identified typologies of cannabis-tobacco users and the level of clinical care they receive (with or without assistance for quitting tobacco).

#### Objective 4 (Second Study)

Validate the “Cannabis Withdrawal Checklist” scale in the Spanish population and explore the relationship between the intensity and duration of craving and abstinence.

#### Objective 5 (Second Study)

Describe and predict the likelihood of cannabis and tobacco abstinence based on consumption patterns (eg, type of consumption), psychological factors (trait and state), and contextual factors (eg, environment, social influences, and activities) while considering structural determinants of inequality.

#### Objective 6 (Third Study)

Investigate the clinical experiences of individuals undergoing treatment for CUD, taking into account their typologies, age, and sex.

In conclusion, the “DuCATA study” aims to achieve 6 objectives across different studies. These objectives involve understanding treatment preferences for CUD through a gamified web app, examining the patterns of cannabis and tobacco use among individuals seeking help, validating assessment scales, and exploring clinical experiences.

## Methods

### Project Design

The project adopts a sequential mixed methods research design encompassing 3 studies. The first study, a qualitative participatory action study aimed at addressing objective 1 by exploring key elements pertinent to the co-design of the gamified web app. The second study is a prospective longitudinal (cohort) study targeting objectives 2 to 5 to quantitatively examine implementation and effectiveness outcomes. The third is a qualitative phenomenological study addressing objective 6 to delve into participants’ experiences within the project. This integrated approach enables a comprehensive investigation into the patterns of consumption and withdrawal syndrome associated with dual cannabis and tobacco use among individuals undergoing cessation treatment for CUD, synthesizing qualitative insights with quantitative findings to provide a holistic understanding of the research objectives.

### Ethical Considerations

The study protocol has been reviewed and approved by the Hospital Universitari de Bellvitge Ethics Committee (PR328/19) and registered on ClinicalTrials.gov (NCT05512091) [27]. The research complies with institutional guidelines and adheres to Law 3/2018 of Personal Data Protection and the General Data Protection Regulation 2016/679 of the European Union. All participants have provided or will provide informed consent before participating in the study. They have been or will be informed of their right to drop out at any time without any negative consequences. For any secondary analyses using existing data, the original consent includes permission for secondary analysis without the need for additional consent. The confidentiality of all information has been or will be ensured, and the research team has adhered to or will adhere strictly to confidentiality rules. Data will be anonymized to protect participant privacy. If data cannot be anonymized, protective measures will be in place to safeguard participant information. Participants do not receive monetary compensation for their participation. Instead, they are thanked and acknowledged for their valuable contribution to the research.

### First Study: Development of a Gamified Web App

#### Objective

The objective is to develop a gamified web app co-designed by the research team and potential users.

#### Method

This study uses a participatory action approach, using focus groups to explore the perspective of potential users and ensure high usability and adherence. Participants include 10 cannabis-tobacco users or former users selected from 3 substance abuse programs (SAP) in Barcelona province, based on purposive sampling and inclusion criteria of 18 years of age or older, undergoing treatment for cannabis use, and owning a smartphone. The focus groups consist of 5 participants, homogeneously grouped by sex and age profiles, with sessions lasting 60 to 90 minutes. Both focus groups and participatory activities are conducted, engaging participants in-person discussions and interactive activities while recording conversations for analysis. Each group is led and coordinated by 3 individuals, including an expert in qualitative research, a web app developer, and a project investigator member.

#### Recruitment

Purposive sampling is used to recruit participants meeting the inclusion criteria. The eligibility criteria are determined by the clinicians of the SAP centers that collaborate in the study.

#### Data Collection Technique

Focus groups and participatory activities, were used, consisting of 2 pilot sessions (where a homemade board game was used to explore participants’ gaming preferences), and 3 participatory sessions (including 2 exploratory sessions with clinicians and former cannabis users to profile this population, and a third session of in-depth interviews to explore into participants’ personal experiences overcoming cannabis use). These sessions were conducted to gather pertinent information for web app design. Each session involved discussions among participants to explore various themes, such as users’ experiences with gamified web apps, expectations and preferences regarding app features, preferred game types, and personal experiences related to cannabis use.

#### Data Analysis

Transcriptions of focus group discussions and participatory activities serve as primary data for analysis, involving familiarization, coding, theme development, data interpretation, and validation. The thematic categorical content analysis will be conducted using ATLAS.ti (ATLAS.ti Scientific Software Development GmbH). Based on these findings, a web app prototype is developed.

#### Design of the Web App

With input from participating groups, an external company experienced in gamified web app design, along with patients and researchers, designs the web app prototype. The prototype incorporates variables of interest in an engaging manner. The same participants involved in co-design will pilot the web app.

### Second Study: Monitoring Patterns of Cannabis and Tobacco Use Among a Cohort of Cannabis Users Searching for Treatment

#### Objective

The aim is to monitor patterns of use of cannabis and tobacco, withdrawal symptoms, abstinence, and other indicators among cannabis users.

#### Method

This study uses a prospective longitudinal follow-up approach. The inception cohort consists of cannabis users attending SAP centers in the province of Barcelona during 2023 to initiate treatment for their disorder.

#### Eligibility for Centers and Clinicians

All SAP coordinators in the province of Barcelona (48 centers) are contacted. We plan to recruit 10 to 14 centers, each recruiting 8 to 12 participants on average.

#### Recruitment

Clinicians recruit individuals who have commenced reducing or quitting cannabis use attending in SAP. Participants are invited to self-manage and self-report their cannabis-tobacco use and symptomatology through a gamified web app and an external software that sends questionnaires regularly (every 6 days) to monitor other behavioral and contextual aspects. Before launching the study, the recruitment protocol is piloted in 3 SAP centers to make necessary adjustments for ease of recruitment by clinicians and increased acceptability among eligible participants.

#### Cohort Members

The cohort members are cannabis users beginning treatment for CUD at SAP centers in Catalonia.

#### Inclusion Criteria

Participants must be older than 18 years of age, daily cannabis users regardless of tobacco consumption pattern or type, in treatment for cannabis use or another substance in an SAP, followed by collaborating clinicians, own a smartphone or computer with an internet connection, commit to daily participation in the web app for 6 weeks, have and use WhatsApp (Meta Platforms) and email, and provide informed consent.

#### Exclusion Criteria

Individuals unable to guarantee 6 weeks of follow-up, those without access to a device for 6 weeks, inability to read and understand Spanish, and moderate or severe cognitive limitations and severe psychopathology, which will be assessed by clinicians during consultation at the time of participant invitation.

#### Sample Size

According to a recent meta-analysis, the probability of cannabis cessation in this patient population treated using diverse therapeutic approaches is relative risks (RR)=1.48 (28). Given its heterogeneity, we will use a more conservative estimate (RR=1.20). Thus, with an alpha risk of 0.05, a beta risk of 0.20 in a bilateral contrast, and accounting for 20% losses to follow-up, 282 participants will be required. Approximately 2000 people start treatment for cannabis use each year in Catalonia [28], making it feasible to recruit 282 participants within a 1-year period.

#### Instruments

##### Gamified Web App

A gamified web app was developed in the first study to prospectively follow-up participants and monitor cannabis and tobacco use, as well as identify withdrawal symptoms (see Table 1). The game of the gamified web app was named DuCATA: Your heroic journey deserves to be told (the original name is “DuCATA: Tu viaje heroico merece ser contado”).

**Table 1:** Variables included in the web app to monitor tobacco and cannabis use.

Frequency	Variable	Description
Every 72 hours	Tobacco use in the last 48 hours	Have you used tobacco in the last 48 hours, with or without cannabis?
Every 72 hours	Cannabis use in the last 48 hours	Have you used cannabis in the last 48 hours?
Every 72 hours	Number of joints	How many joints have you consumed in the last 48 hours?
Every 72 hours	Cannabis reduction	Compared to 2 days ago, have you reduced your cannabis use?
Every 72 hours	Cannabis withdrawal checklist	Have you experienced any of these symptoms in the last 48? Shakiness or tremulousness Depressed mood Decreased appetite Nausea Irritability Sleep difficulty Sweating Craving to smoke marijuana Restlessness Nervousness or anxiety Increased aggression Headaches Stomach pains Strange dreams Increased anger

##### Monitoring Software

The monitoring software was designed to record participants’ recruitment, obtain their baseline information, and periodically send additional questionnaires to assess participant behavior, contextual factors, and so forth (see Table 2). This software was created to carry out the study, and we have named it DuCATA software.

**Table 2:** Baseline and follow-up variables regarding sociodemographic characteristics, tobacco, cannabis use, attitudes, social norms, and motivation to quit of the participants (variables included in the monitoring software).

Variables				Temporality						
				Baseline before acceptance	Baseline after acceptance	6 days software	18 days software	Final software		
**Dependent variables**										
		**Cannabis addiction**		✓	✓	✓		✓		
			Cannabis use pattern	✓	✓	✓				
			Dependency severity		✓	✓				
			Cannabis type and way of use		✓		✓			
			Cannabis withdrawal symptoms		✓	✓				
		**Addiction to tobacco**		✓	✓			✓		
			Tobacco use pattern		✓	✓				
			Nicotine dependence		✓	✓				
		Alcohol addiction			✓	✓				
		**Attitudes**			✓					
			Psychoemotional factors		✓		✓			
			Social environment		✓		✓			
		**Subjective perception of norms**			✓		✓			
			Beliefs and expectations of consumption and treatment					✓		
		Self-efficacy			✓	✓				
		Level of approach in smoking or cannabis received			✓			✓		
		**Motivation**				✓				
			Cannabis dropout							
			Tobacco dropout			✓				
**Independent variables**										
	Recruiting center			✓						
	Sociodemographic variables				✓					
	Basic mental and physical health measures				✓		✓			
	Family environment or place of residence				✓					

### Primary Quantitative Effectiveness Outcomes of the Web App in Monitoring Behavioral Changes

#### End Points

The end points are (1) variables reported every 3 days during 6 weeks within the web app and (2) variables collected in the external software at baseline and every 6 days.

#### Cannabis Use

Cannabis use is determined based on the (1) consumption of cannabis in the last 48 hours (yes or no), reduction of cannabis in the last 48 hours (yes or no), and cannabis abstinence or reduction requiring a cannabis withdrawal test and (2) cannabis product used (hashish, marijuana, etc), mode of administration (smoking, snorting, ingestion, etc), frequency of cannabis use in the last month and week, type of use (alone, accompanied, and both), and reasons for use (addiction, recreational, social, sleeping, etc).

#### Tobacco Use

Tobacco use is determined based on (1) tobacco consumption in the last 48 hours (yes or no) and (2) pattern of tobacco use—type of tobacco products (manufactured cigarettes, roll-your-own, electronic cigarettes, heated tobacco, or similar), frequency of use (daily and occasional), number of units (cigarettes, etc), and nicotine dependence measured using the Heavy Smoking Index (0 to 6), classified as low (0-2), moderate (3-4), and high (5-6) [29,30].

#### Abstinence From Cannabis, Tobacco, and Both

If abstinent of both substances we will ask for a CO measurement test (performed by their clinician and introduced in the software; yes or no).

#### Cannabis-Tobacco Users’ Classification

Cannabis-tobacco users will be classified as concurrent use, sequential concurrent use, asynchronous concurrent use, and exclusive cannabis use.

#### ASE Model variables

The ASE model variables are attitudes, subjective norms, and self-efficacy.

#### Attitudes

Motivation to quit was measured on a Likert scale, psychological factors (states of change or readiness to quit cannabis and tobacco and psychological distress) [31]. Psychological distress was measured by using the General Health Questionnaire (GHQ-12) [32].

#### Self-Perceived Health Status

The self-perceived health status will be assessed based on “How would you say your general health is?” (excellent, very good, good, fair, fair, or poor).

#### Self-Efficacy

Self-efficacy is defined as the individual’s confidence to quit cannabis and tobacco [33]. This will be assessed by asking participants, “To what extent do you feel capable of quitting cannabis consumption?” using a Likert scale ranging from 0 to 10 (0= very low to 10= very high).

#### Independent Variables

Sociodemographic variables are (baseline) sex, age, education level, employment status, and occupation. Mental and physical health measures are mental illness presence and comorbidities. Centers are where care is received. The family environment is cohabitation, socioeconomic level, and so forth.

#### Implementation Outcomes

To test the acceptability, appropriateness, and feasibility besides using the qualitative methodology explained, we will quantitatively measure these dimensions among all the participants enrolled in the cohort. We will use the scale developed by Weiner et al [34] that consists of 12 items scored from 1 (completely disagree) to 5 (completely agree). This evaluation will be conducted at the end of the follow-up period (6 weeks), and we will collect this information using a customized questionnaire administered via phone call or text message. Additionally, we will assess other implementation outcomes at the end of study 2 by gathering data on (1) acceptability—the number of individuals who accept or decline to participate in the study; (2) fidelity or adherence—the duration of participants’ usage of the web app, time devoted to playing, number of completed sessions, and so forth; and (3) satisfaction—participants’ opinions about the proposed web app. These variables will be measured using the metadata of the web app (encrypted).

#### Recruitment Procedure

Clinicians verify participant eligibility using a checklist of inclusion and exclusion criteria. They provide oral and written information to potential participants through a leaflet containing study details and information about the study sponsors. Potential participants are informed about the study objectives, procedures, potential risks, benefits, confidentiality measures, and their right to withdraw at any time. This process ensures that only eligible participants who meet the criteria are enrolled in the study.

Additionally, clinicians show a short video explaining the purpose of the research and soliciting participants’ collaboration (available on YouTube [35]). If participants agree to participate, they sign the informed consent and then complete a questionnaire with brief personal information (telephone number, email, name, and national identity document). Subsequently, participants register on the web app and engage with the game daily.

#### Statistical Methods

For independent variables, a descriptive analysis will be conducted, including frequencies, percentages, and their 95% CIs. To assess effectiveness measures such as changes in cannabis-tobacco use, withdrawal syndrome, and treatment adherence, frequencies and percentages will be described for baseline and main assessment end points according to the four patterns of cannabis use and they are (1) concurrent, (2) sequential concurrent, (3) asynchronous concurrent, and (4) exclusive cannabis. Incidence rates of abstinence and symptomatology will be calculated for the same assessment end points across the 4 patterns. The RR of quitting or reducing cannabis and tobacco, as well as quit attempts for both substances (95% CI), adjusted for independent variables, and initial consumption pattern, will be calculated using Cox or Poisson robust regression models. Predictive analysis techniques including logistic regression and random forest will be used to model the probability of events occurring based on other factors. Time series analysis will be used to observe consumption trends during the follow-up, and survival analysis of recurring events will be conducted to observe abstinence or reduction processes among participants.

For all analyses, SPSS (IBM Corp) for Windows version 21, Python (version 3; Python Software Foundation), R (version 4.0.3; R Core Team), and scikit-learn will be used. The level of significance will be set at 5% (*P*<.05).

### Third Study: Assessment of the Acceptability of the Web App

#### Objective

The objective is to evaluate the acceptability of the web app and investigate participant and clinician experiences during study 2, focusing on implementation outcomes.

#### Method

The methodology used is a qualitative phenomenology study using participatory groups.

#### Sample

Participants will be selected from study 2 cohort members based on their web app usage experience. A total of 4 groups comprising 5-7 participants each are anticipated.

#### Recruitment

Purposive sampling will be used, targeting individuals meeting the inclusion criteria and having engaged with the web app. Homogeneity criteria will be applied to ensure diversity across fidelity levels and gender—group 1: high fidelity male users; group 2: low fidelity male users; group 3: high fidelity female users; and group 4: low fidelity female users.

#### Main Themes

The main themes are exploration of barriers and opportunities encountered during web app usage, assessment of the use of web app monitoring for cannabis or tobacco use and mood, and identification of preferred and disliked features of the web app.

#### Data Collection Technique

Focus groups are expected to last between 60 and 90 minutes. Conversations will be recorded to facilitate transcription and analysis while ensuring confidentiality. Each group will be facilitated by 2 individuals, including an expert in qualitative research and a project investigator.

#### Data Analysis

Transcribed data will undergo analysis involving familiarization, coding, theme development, interpretation, and validation; the thematic categorical content analysis will be conducted using ATLAS.ti. Insights garnered will inform the refinement of a web app prototype.

## Results

By June 2024, significant progress had been achieved in the Dual use of Cannabis and Tobacco Monitored through a Gamified Web app (DuCATA_GAM-CaT) project.

### First Study: Development of a Gamified Web App

Study 1, focusing on qualitative investigation through participatory focus groups, was completed, providing valuable insights into the profile of cannabis users eligible for the project.

Additionally, an initial prototype of the gamified web app has been developed, representing a critical advancement in addressing cannabis-tobacco withdrawal symptoms among individuals with CUD attending SAP.

### Second Study: Monitoring Patterns of Cannabis and Tobacco Use Among a Cohort of Cannabis Users Searching for Treatment

**Figure 2:**
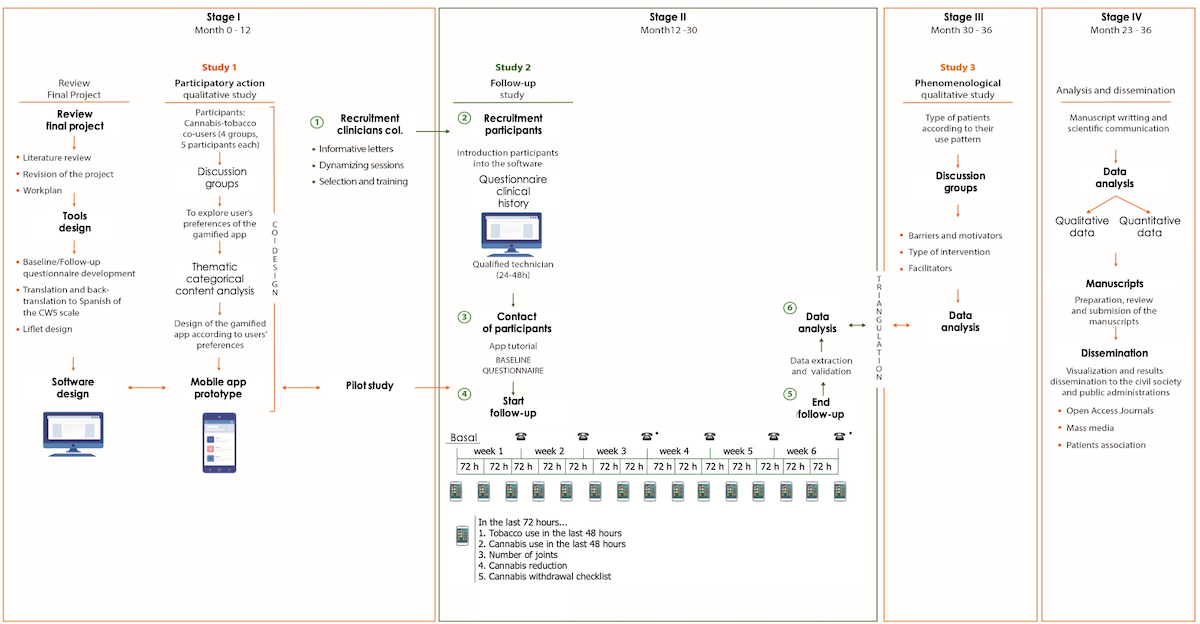
Study timeline.

Recruitment efforts have commenced across 10 centers, with 74 participants enrolled to date. We anticipate reaching our target enrollment of 150 participants shortly (see Figure 2). This longitudinal prospective study will track participants’ cannabis-tobacco usage patterns, types and frequencies of tobacco use, nicotine dependence levels, withdrawal symptoms, psychoemotional factors, and motivation to quit both substances over a 6-week period.

### Third Study: Assessment of the Acceptability of the Web App

Once study 2 is finished, we will start this qualitative study, which is scheduled for January 2025.

## Discussion

### Principal Findings

To address the objective of the study, we aimed to characterize the patterns of cannabis and tobacco use among individuals initiating cannabis treatment at SAP centers, while also monitoring cannabis withdrawal syndrome in both tobacco users and nonusers. Our primary objectives include acquiring knowledge and establishing the groundwork for a clinical intervention program theory.

### Main Findings

Our study will provide insights into the diverse patterns of cannabis and tobacco use among participants entering treatment at SAP centers. We will identify their symptomatology if cannabis withdrawal syndrome appears, whether in combination with tobacco use or alone, which will help us determine the overlap of the symptoms.

Additionally, the mixed methods design of our study will allow for the triangulation of results from various perspectives, facilitating a comprehensive understanding of the phenomena under investigation.

### Comparison to Prior Work

We will compare our results with the scarce literature on the topic. We will not only be able to understand the complexity of use and withdrawal symptoms but also explore the use of a gamified app for monitoring several variables of interest.

### Strengths and Limitations

A key strength of our study lies in its methodological rigor, combining qualitative exploration with quantitative measurement. However, the study is limited by its observational nature and the potential for self-report biases in substance use reporting.

### Future Directions

Moving forward, these findings can inform the development of targeted intervention strategies aimed at cannabis cessation, whether used exclusively or in combination with tobacco. Future studies could further explore specific intervention protocols and assess their efficacy in diverse clinical settings.

### Conclusions

In conclusion, our study contributes valuable insights into the complex interplay between cannabis and tobacco use behaviors and withdrawal symptoms. These findings lay a foundation for future research and intervention efforts aimed at addressing CUD within substance abuse treatment programs.

## Abbreviations

ASE: Attitudes, Social Influences, and Efficacy
CUD: cannabis use disorder
DSM-5: Diagnostic and Statistical Manual of Mental Disorders (Fifth Edition)
DuCATA: Dual users of Cannabis and Tobacco
DuCATA_GAM-CaT: Dual use of Cannabis and Tobacco Monitored through a Gamified Web app
EMA: Ecological Momentary Assessment
GHQ-12: General Health Questionnaire
RR: relative risks
SAP: substance abuse programs

## Data Availability

The data sets generated and analyzed during this study are available from the corresponding author on reasonable request.

## References

[1] (2019). World drug report 2019. United Nations.

[2] Ministerio de Sanidad, Gobierno de España (2019). Encuesta Nacional de Salud de España 2019. Ministerio de Sanidad.

[3] Schwitzer T, Gillet C, Bisch M, Di Patrizio P, Schwan R, Laprevote V (2016). [Co-occurrent cannabis and tobacco uses: clinical knowledge and therapeutic prospects]. Therapie.

[4] Schauer GL, Peters EN (2018). Correlates and trends in youth co-use of marijuana and tobacco in the United States, 2005-2014. Drug Alcohol Depend.

[5] Casajuana C, López-Pelayo H, Mercedes Balcells M, Miquel L, Teixidó L, Colom J, Gual A (2017). Working on a standard joint unit: a pilot test. Adicciones.

[6] Schauer GL, Rosenberry ZR, Peters EN (2017). Marijuana and tobacco co-administration in blunts, spliffs, and mulled cigarettes: a systematic literature review. Addict Behav.

[7] Lemyre A, Poliakova N, Bélanger RE (2019). The relationship between tobacco and cannabis use: a review. Subst Use Misuse.

[8] Davis ML, Powers MB, Handelsman P, Medina JL, Zvolensky M, Smits JAJ (2015). Behavioral therapies for treatment-seeking cannabis users: a meta-analysis of randomized controlled trials. Eval Health Prof.

[9] Bahji A, Stephenson C, Tyo R, Hawken ER, Seitz DP (2020). Prevalence of cannabis withdrawal symptoms among people with regular or dependent use of cannabinoids: a systematic review and meta-analysis. JAMA Netw Open.

[10] Herrmann ES, Weerts EM, Vandrey R (2015). Sex differences in cannabis withdrawal symptoms among treatment-seeking cannabis users. Exp Clin Psychopharmacol.

[11] American Psychiatric Association, DSM-5 Task Force (2013). Diagnostic and Statistical Manual of Mental Disorders: DSM-5™ (5th ed.).

[12] Allsop DJ, Norberg MM, Copeland J, Fu S, Budney AJ (2011). The cannabis withdrawal scale development: patterns and predictors of cannabis withdrawal and distress. Drug Alcohol Depend.

[13] Allsop DJ, Copeland J, Norberg MM, Fu S, Molnar A, Lewis J, Budney AJ (2012). Quantifying the clinical significance of cannabis withdrawal. PLoS One.

[14] Allsop DJ, Dunlop AJ, Saddler C, Rivas GR, McGregor IS, Copeland J (2014). Changes in cigarette and alcohol use during cannabis abstinence. Drug Alcohol Depend.

[15] Bonnet U, Specka M, Stratmann U, Ochwadt R, Scherbaum N (2014). Abstinence phenomena of chronic cannabis-addicts prospectively monitored during controlled inpatient detoxification: cannabis withdrawal syndrome and its correlation with delta-9-tetrahydrocannabinol and -metabolites in serum. Drug Alcohol Depend.

[16] Struble CA, Ellis JD, Cairncross M, Lister JJ, Lundahl LH (2019). Demographic, cannabis use, and sepressive correlates of cannabis use consequences in regular cannabis users. Am J Addict.

[17] Livne O, Shmulewitz D, Lev-Ran S, Hasin DS (2019). DSM-5 cannabis withdrawal syndrome: demographic and clinical correlates in U.S. adults. Drug Alcohol Depend.

[18] Gonzalez-Cuevas G, Martin-Fardon R, Kerr TM, Stouffer DG, Parsons LH, Hammell DC, Banks SL, Stinchcomb AL, Weiss F (2018). Unique treatment potential of cannabidiol for the prevention of relapse to drug use: preclinical proof of principle. Neuropsychopharmacology.

[19] Anthony JC, Lopez-Quintero C, Alshaarawy O (2017). Cannabis epidemiology: a selective review. Curr Pharm Des.

[20] de Vries H, Backbier E (1994). Self-efficacy as an important determinant of quitting among pregnant women who smoke: the phi-pattern. Prev Med.

[21] Wray JM, Gass JC, Tiffany ST (2013). A systematic review of the relationships between craving and smoking cessation. Nicotine Tob Res.

[22] Serre F, Fatseas M, Debrabant R, Alexandre J, Auriacombe M, Swendsen J (2012). Ecological momentary assessment in alcohol, tobacco, cannabis and opiate dependence: a comparison of feasibility and validity. Drug Alcohol Depend.

[23] Connor JP, Symons M, Feeney GFX, Young RM, Wiles J (2007). The application of machine learning techniques as an adjunct to clinical decision making in alcohol dependence treatment. Subst Use Misuse.

[24] Lee MR, Sankar V, Hammer A, Kennedy WG, Barb JJ, McQueen PG, Leggio L (2019). Using machine learning to classify individuals with alcohol use disorder based on treatment seeking status. EClinicalMedicine.

[25] Gentry SV, Gauthier A, L'Estrade Ehrstrom B, Wortley D, Lilienthal A, Tudor Car L, Dauwels-Okutsu S, Nikolaou CK, Zary N, Campbell J, Car J (2019). Serious gaming and gamification education in health professions: systematic review. J Med Internet Res.

[26] Serre F, Fatseas M, Denis C, Swendsen J, Auriacombe M (2018). Predictors of craving and substance use among patients with alcohol, tobacco, cannabis or opiate addictions: commonalities and specificities across substances. Addict Behav.

[27] Characterization of the pattern of consumption and withdrawal syndrome from dual cannabis and tobacco use (DuCATA_GAMCaT). National Institutes of Health.

[28] (2021). Tratamientos por drogodependencia. Por tipo de droga, sexo, grupos de edad, situación laboral y nivel de instrucción. Instituto de Estadística de Cataluña.

[29] Kozlowski LT, Porter CQ, Orleans C, Pope MA, Heatherton T (1994). Predicting smoking cessation with self-reported measures of nicotine dependence: FTQ, FTND, and HSI. Drug Alcohol Depend.

[30] Heatherton TF, Kozlowski LT, Frecker RC, Rickert W, Robinson J (1989). Measuring the heaviness of smoking: using self-reported time to the first cigarette of the day and number of cigarettes smoked per day. Br J Addict.

[31] DiClemente CC, Prochaska JO, Fairhurst SK, Velicer WF, Velasquez MM, Rossi JS (1991). The process of smoking cessation: an analysis of precontemplation, contemplation, and preparation stages of change. J Consult Clin Psychol.

[32] Rocha KB, Pérez K, Rodríguez-Sanz M, Borrell C, Obiols JE (2011). Propiedades psicométricas y valores normativos del general health questionnaire (GHQ-12) en población general española. Int J Clin Health Psychol.

[33] Bandura A (1986). Social Foundations of Thought and Action: A Social Cognitive Theory.

[34] Weiner BJ, Lewis CC, Stanick C, Powell BJ, Dorsey CN, Clary AS, Boynton MH, Halko H (2017). Psychometric assessment of three newly developed implementation outcome measures. Implement Sci.

[35] DuCATA: Tu viaje heroico merece ser contado. YouTube.

